# Clinical Lectures on Albuminuria

**Published:** 1889-03

**Authors:** 


**Clinical Lectures on Albuminuria.** By THOMAS GRAINGER STEWART, M. D., Edin.; Fellow of the Royal College of Physicians of Edinburgh; M. D. Honoris Causa Royal University of Ireland; Hon. Fellow King and Queen's College of Physicians in Ireland; Physician-in-Ordinary to Her Majesty the Queen, for Scotland; Professor of the Practice of Physic and of Clinical Medicine in the University of Edinburgh. 8vo, pp. 250. New York: William Wood & Company. 1888.

Since the publication of the first edition of his practical treatise on Bright's Disease of the Kidneys, in 1868, Dr. Thomas Grainger Stewart has everywhere been recognized as authority on that subject. His book passed to its second edition in 1871, since which time we have become accustomed to consult it when needing aid on the subject of the kidneys.

This new work is an embodiment of his lectures delivered in the University of Edinburgh during the past two years. Some of them have been published in the journals from time to time, so that our readers are doubtless familiar with their scope; but now they have been collected into book form, making most convenient reference, and assuring them that permanency which belongs to a library volume.

A complete table of the authors referred to in the text, and a comprehensive index, makes it a most convenient book of reference. It is entirely unnecessary to critically analyse the contents of this volume, for the reasons already given. It is quite necessary that all who would keep abreast of the literature of diseases of the kidneys, should possess it. The publishers deserve special commendation for the quality and style of the volume.

